# Improving Alzheimer’s Disease Classification in Brain MRI Images Using a Neural Network Model Enhanced with PCA and SWLDA

**DOI:** 10.3390/healthcare11182551

**Published:** 2023-09-15

**Authors:** Irshad Ahmad, Muhammad Hameed Siddiqi, Sultan Fahad Alhujaili, Ziyad Awadh Alrowaili

**Affiliations:** 1 Department of Computer Science, Islamia College, Peshawar 25000, KPK, Pakistan; 2 College of Computer and Information Sciences, Jouf University, Sakaka 2014, Aljouf, Saudi Arabia; 3 College of Applied Medical Sciences, Jouf University, Sakaka 2014, Aljouf, Saudi Arabia; 4 Department of Physics, College of Science, Jouf University, Sakaka 2014, Aljouf, Saudi Arabia

**Keywords:** medical imaging, neuroimaging, Alzheimer’s disease, PCA, SWLDA, ANN, feature extraction, feature selection, classification, healthcare

## Abstract

The examination of Alzheimer’s disease (AD) using adaptive machine learning algorithms has unveiled promising findings. However, achieving substantial credibility in medical contexts necessitates a combination of notable accuracy, minimal processing time, and universality across diverse populations. Therefore, we have formulated a hybrid methodology in this study to classify AD by employing a brain MRI image dataset. We incorporated an averaging filter during preprocessing in the initial stage to reduce extraneous details. Subsequently, a combined strategy was utilized, involving principal component analysis (PCA) in conjunction with stepwise linear discriminant analysis (SWLDA), followed by an artificial neural network (ANN). SWLDA employs a combination of forward and backward recursion methods to choose a restricted set of features. The forward recursion identifies the most interconnected features based on partial *Z*-test values. Conversely, the backward recursion method eliminates the least correlated features from the same feature space. After the extraction and selection of features, an optimized artificial neural network (ANN) was utilized to differentiate the various classes of AD. To demonstrate the significance of this hybrid approach, we utilized publicly available brain MRI datasets using a 10-fold cross-validation strategy. The proposed method excelled over existing state-of-the-art systems, attaining weighted average recognition rates of 99.35% and 96.66%, respectively, across all the datasets.

## 1. Introduction

Medical imaging data are showing rapid growth, owing to advancements in hardware technology, population growth, reduced costs, and the recognition of the valuable applications of imaging modalities [1]. Magnetic resonance imaging (MRI) holds significant prominence in Alzheimer’s disease (AD) research due to its noninvasive characteristics and minimal patient discomfort [2]. Additionally, MRI provides exceptional spatial resolution and effective contrast, rendering it an invaluable asset [3,4,5,6]. The accurate classification of clinical findings in AD using MRI is a critical and fundamental step in the treatment process. However, this classification is susceptible to multiple misclassifications owing to the significant similarity evident among brain images [7].

The initial stages of AD display a gradual onset, posing a challenge for detection. Nevertheless, as the disease advances, it markedly affects daily functionality and causes irreversible brain damage. This imposes a substantial burden on both the families of patients and healthcare systems [8]. Currently, there is a lack of functional clinical strategies for the prevention or treatment of AD, with existing medications only capable of slowing the progression of the disease. Consequently, the early diagnosis of AD has emerged as one of the most significant concerns confronting both the medical field and society as a whole [9,10].

The latest model was suggested by [11], where the segmentation and classification were performed using transfer learning and deep learning techniques on MRI images. For this purpose, they specifically utilized those images which were segmented through gray matter. Moreover, in their model, they employed a former-trained convolutional neural network (CNN) model for training and validation followed by transfer learning. Similarly, an integrated approach has been proposed by [12] based on, respectively, the classification features and iterative features. In this approach, four methods were utilized: among them, three methods were used to diminish the interaction from the classification data. Furthermore, from a set of training data, a homogenous sub-group was created. The third approach, which was a combination of two methods, assessed against the entire baseline data of AD for two multilevel classification works. The authors of [13] developed and validated a method that was based on deep learning, which predicted AD and slight rational damage using brain MRI. Further, CNNs were utilized on weighted MRIs to evaluate the accomplishment of their approach.

On the other hand, an ensembled approach was designed [14] which was based on various optimum techniques such as particle swarm optimization, genetic algorithm, cuckoo search, and grey wolf optimization. In the first phase of this approach, the brain MRI is segmented into several regions, which require the MRI images from AD patients. Then, the performance of segmentation is assessed under the presence of segmented regions along with ground truth images, which is used to calculate various kinds of matrices like feature and structure similarity indexes, dice similarity, etc., and the developers have reported a 95% successful classification rate. Another state-of-the-art automatic deep convolution neural-based system was proposed by [15], which was coupled with the AlexNet framework for the categorization of AD against MRI images. They validated their model on different types of datasets like very mild demented, non-demented, moderately demented, and mildly demented MRI datasets, respectively. Likewise, a deep-learning-based method has been suggested [16] to resolve the complexity and classification issues involved in using structural magnetic resonance images, and the researchers have reported good performance. Existing works have revealed that cortical depth and the capacity of the hippocampus play a significant part in the development of AD [17]. Additionally, most of these systems have limited and controlled experiments to validate them.

Consequently, this study proposes an adaptive model for the classification of AD using MRI images. Our research comprised the following:

- The presence of noisy and environmental factors could potentially reduce the system’s accuracy; thus, to mitigate the influence of these factors, we incorporated an adaptive averaging filter during preprocessing.
- Subsequently, we utilized PCA to extract the most relevant features from the MRI images. PCA reduces complex data by projecting them onto a lower-dimensional space, retaining a maximum amount of their inherent variability.
- After feature extraction, it is possible that there is some redundancy within the feature set. To address this, we introduced a novel approach called SWLDA, which is designed to identify optimal features. This algorithm can discern the most distinctive features from a variety of disease-related MRI frames, to ultimately select the most advantageous features. SWLDA employs a combination of forward and backward recursion techniques to choose a limited number of features. The forward recursion approach identifies the most interconnected features based on partial *Z*-test values. Conversely, the backward recursion method eliminates the least correlated features from the same feature space. Following the feature selection process, we utilized an artificial neural network (ANN) to classify the multi-level categories of AD.
- A comprehensive set of experiments, incorporating diverse factors, was conducted to demonstrate the efficacy of the proposed method using a publicly accessible MRI dataset [18]. Furthermore, a thorough comparative analysis is provided to assess the performance of the proposed approach in comparison to contemporary existing methods.

The remainder of this article has the following structure. In Section 2, we comprehensively present the state-of-the-art studies along with their shortcomings, while in Section 3, the proposed approach is presented in detail. In Section 4, the corresponding dataset and experimental setup are described. Section 5 reports and discusses the results. Lastly, the article is summarized in Section 6 with some future directions.

## 2. Literature Review

Alzheimer’s disease (AD) is a prominent brain disorder, often observed in older individuals. The primary factor underlying this condition is the deterioration of memory and cognitive function, with advanced stages of AD leading to significant memory loss [19].

A traditional multimodal fusion strategy was introduced by [20,21] that employs a discrete wavelet transform (DWT), a mathematical method, for data analysis. To enhance the performance of this approach, transfer learning is applied via the VGG16 neural network, which is pretrained. The ultimate fused image is reconstructed using an inverse discrete wavelet transform (IDWT). The fused images undergo classification using a pre-trained vision transformer. However, this approach has higher feature dimensions for training, due to which, it is much more expensive and suffers from over-fitting problems [22].

Likewise, the efficiency of the Pareto-optimized VGG model was investigated in comparison to conventional VGG variations. This investigation aimed to assess the capability of these deep learning models in extracting significant features from MRI and PET data, as demonstrated in their ability to extract crucial features [23]. However, this model is unable to identify alterations in the brain networks of patients with mildly impaired functional working brain networks [24]. An accurate system was proposed by [25] that was based on transfer learning for the classification of AD at different stages. This approach categorizes normal, early-mild, late-mild, and AD brains. For this purpose, they employed the segmentation of tissues to extract the gray matter from the standard MRI dataset of AD. While freezing the various kinds of features, we used the gray matter to tune the architecture. However, this technique may not be effective if the classification layer is not able to distinguish between the different categories for a specific issue [26]. A recent study conducted by researchers [27] employed a statistical analysis to predict the onset of AD using brain MRI. They employed a range of classifiers, including random forest, decision tree, gradient boosting, support vector machine, and voting-based methods, to identify the best parameters for predicting AD. They employed a range of classifiers, including random forest, decision tree, gradient boosting, support vector machine, and voting-based methods, to identify the best parameters for predicting AD. They showed good performance on a publicly available standard dataset of brain MRI images. Most of these methods have their limitations; however, these classifiers depend upon the traditional machine learning classifiers which do not allow for hyperparameter modification. This characteristic might decrease the performance and rate of identification of AD [28]. Similarly, a CNN-based approach was developed by [15] to predict AD and mild cognitive disorders, relying solely on single cross-sectional MRI images. The proposed CNN was employed on three-dimensional T1-weighted images to distinguish AD and mild cognitive disorders, and they reported 98% accuracy. Although CNNs have achieved significant success in identifying AD, there are several challenges arising from the limited availability of medical data and their potential application in such domains [29].

Alternatively, the latest ensemble architecture was developed by [30] for the identification of AD using brain MRI images. The architecture extracted various kinds of features from the MRI images using a CNN. The classification was then performed by employing a random forest (RF) algorithm, which was compared to state-of-the-art methods. However, several issues have been raised, including the shortage of medical data and the potential scope of CNNs in such domains [26]. Furthermore, for data that include categorical variables with varying levels, RF can exhibit bias towards those features with a higher number of levels [31]. A deep-learning-based model was suggested by [32] for the diagnosis of AD using a brain MRI dataset. In this model, the authors utilized ResNet with 50 layers and DenseNet with 169 layers, which were used for the classification of AD into non-dementia, very mild dementia, mild dementia, and moderate dementia. Both of the methods showed significant performance on brain MRI datasets. The methods utilized in this approach, such as the ResNet, are significantly comparable to the DenseNet; however, they also exhibit key differences [33].

A very recent work, undertaken by [34], utilized deep learning strategies with a brain MRI dataset. In this approach, the authors performed multilevel classification using transfer learning coupled with VGG-16 and Fastai to identify the various kinds of diseases, including AD. However, this approach has an overfitting problem. Likewise, Fan et al. [35] proposed an SVM-based model for classifying AD using structural brain MRI. In this work, the SVM model was combined with MRI data to achieve accurate AD predictions. However, SVM has a common limitation in that it cannot choose the appropriate kernel function. A state-of-the-art system was designed by [36] for the multilevel categorization of AD using brain MRI, which was based on a Siamese CNN. They also employed a triple-loss function for the illustration of corresponding input MRI slices as k-dimensional insertion. In their experimental setting, they utilized pre-trained and post-trained CNNs to show the significance of the system. However, the classification rate for some diseases in this model is not sufficiently high to provide actual decision-making support [37]. In contrast, a volumetric-CNN-based architecture has been designed by [38] for binary classification, including AD. The system employes convolutional-autoencoder (CAE)-based, unsupervised and supervised learning to improve the recognition rate of the system. However, this approach is limited by the fact that it requires the system to first identify discriminative landmark positions in MRI slices; hence, the signification of this architecture is influenced [39].

Another state-of-the-art ensembled approach was suggested by [40] for the identification of AD using brain MRI slices. They integrated the pre-trained CNN with ResNet-50 to automatically extract the various kinds of features from MRI images. They assessed their performance using SVM, random forest, and conservative Softmax and thereby achieved good classification accuracy [40]. However, random forests can be overly influenced by features that have more levels for data with categorical variables [31]. Additionally, a common concern with SVM is that it cannot choose the suitable kernel function Furthermore, the CNN-Softmax model cannot optimally determine some of the factors such as the size of the layers, the number and kernels of the layers, respectively [41].

Consequently, we have depicted an effective and accurate feature selection technique for the MRI classification system, due to which the system precisely categorizes different types of brain diseases. The suggested method selects only a limited feature through forward together with backward recursion methods. In the forward recursion method, the most interrelated features are identified based on the values of the partial Z-test, while, in the backward recursion method, the slightest correlated features are removed from the same feature space. In both cases, the Z-test values are assessed using the provided disease labels. The proposed method has a notable strength in its ability to efficiently and effectively identify localized features.

## 3. Suggested Hybrid Scheme

**Figure 1:**
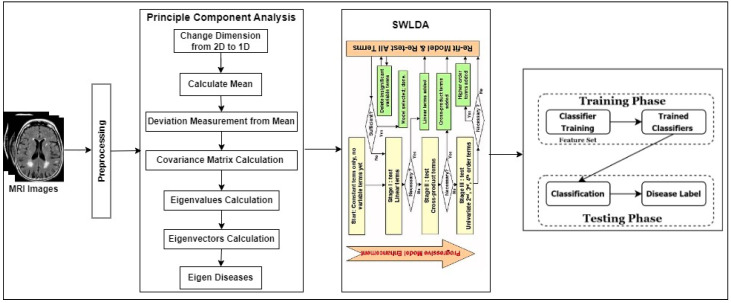
The flow diagram of the suggested approach.

The entire concept of the approach is prescribed in Figure 1.

### 3.1. Preprocessing (Image Normalization)

For image normalization, we employed an averaging filter, where each point in the corresponding image such as *I* (*x*, *y*) is derived from the average value of the surrounding pixel of (*x*, *y*) in the corresponding image. For example, the following 3 × 3 mask is utilized for the surrounding pixels.

191919191919191919

Hence, the corresponding pixel values are enlarged via 1/9, added, and the result is placed in the respective subsequent MRI image. The aforementioned mask continuously moves, pixel-by-pixel, over the whole MRI image. This process will continue until the last modification of the pixel, which means the processed image of the MRI is complicated with this corresponding window, which is also referred to as a symmetrical filter. Please refer to [42] for more details on averaging filters.

### 3.2. Feature Extraction and Dimension Reduction Using Principal Component Analysis (PCA)

**Figure 2:**
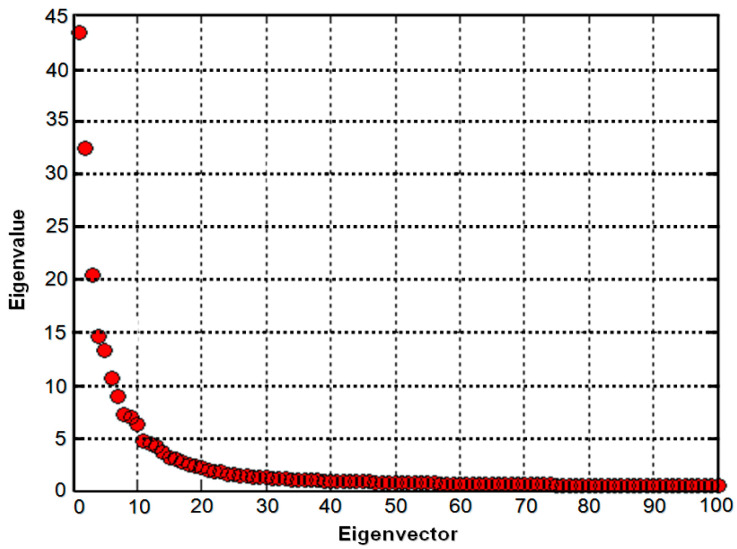
The eigenvectors corresponding to the top 150 eigenvalues.

After the preprocessing step, the PCA was utilized to extract the most significant global features. This approach facilitates the reduction of a complex dataset by mapping it onto a low-dimensional space. The objective is to preserve as much information as possible while minimizing the impact of random variations. PCA produces the prominent linear minimal squares fragmentation of a training set. It does not make any suppositions about the data distribution. It calculates the actual data along with the low-dimension features that provide an economical description of the data. The main purpose of employing PCA in this work is to convey the large, one-dimensional pixels’ vector which is generated from the two-dimensional MRI slice into the dense principal coefficients of the feature space, which is named the prediction of the eigenspace. Moreover, the essential task of PCA is to calculate the eigenvectors of the covariance data matrix, and then by the integration of a certain higher eigenvectors, the estimation is performed. We selected the top 150 eigenvectors with their respective eigenvalues presented in Figure 2, where a total of 6400 MRI images were tackled for PCA.

Please refer to [43] for more details on principal component analysis.

### 3.3. Developed Feature Selection Technique

This section discusses, a highly acclaimed linear classification technique known as a Fisher linear discriminant, used for the partition amongst the two classes [44]. While the Gaussian distribution technique can be applied to two classes taking a similar coefficient, FLD serves as a superior robust classifier that computes optimal partition among the classes. FLD can be compared with recursion techniques such as the least square technique, and can also predict the masses of their features in the domain of binary jobs as follows.

(1) $$\hat{L}={\left(M^{t}M\right)}^{-1}M^{t}y$$

where *L* represents the class label and *M* represents the pragmatic feature vector matrix, and *y* is the class label. FLD shows strong classification performance, but only for data that are linear. However, to deal with non-linear classification challenges, we propose a novel idea based on the use of SWLDA. This technique has been verified using the P300 Speller response [45]. The added capability of SWLDA in comparison with FLD is that the latter works side by side to reduce feature space and eliminate irrelevant information.

SWLDA uses a dual parallel approach involving two algorithms, namely forward and backward for the selection of best features. The model achieved the most significant value with “*p*-value < 0.15” without an initial model at the beginning. The forward algorithm was employed to enter values, followed by the backward algorithm to eliminate unnecessary variables, specifically those with a “*p*-value > 0.2”. This process iterates until a pre-planned environment is satisfied, limiting the evolve function to 125 features.

The method of regression involves the selection of the finest variables like *C* and then moving on to add further $C_{s}$ expressively. The procedure of adding the latest record and the value selections depends upon the Z-test value that determines the order of entry. A comparison is then made between the limited Z-value and the selected value. During the entire procedure, the forward method is employed. Conversely, a backward method known as backward deletion is used in the deletion procedure, where the testing is calculated that is present in the backlog. If the conclusive test has the least value (*V_L_*) then it is compared with the pre-picked value (*P_p_*).

- If $V_{L}<P_{p}$, then restart the calculation of the *F*-test.
- Otherwise, if $V_{L}>P_{p}$, then accept the regression equation.

**Figure 3:**
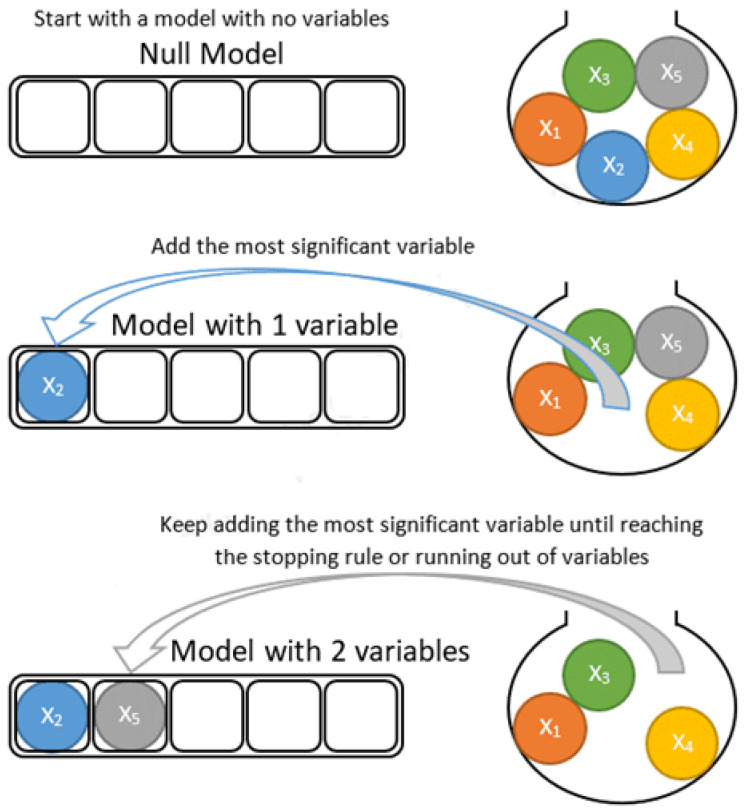
The working diagram of the forward method along with some variables [46].

**Figure 4:**
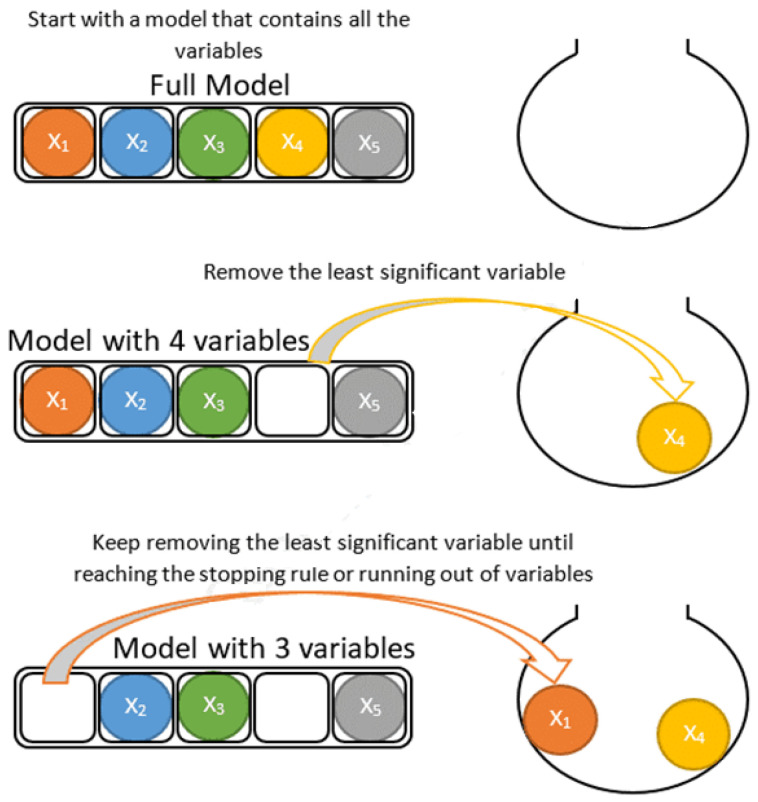
The working diagram of the backward method along with some variables [46].

Based on the proposed approach, the method is constructed to prescribe the iterations. Independent variables are automatically selected in each iteration. Stepwise linear discriminant analysis (SWLDA) depends upon the stepwise regression that employs forward and backward methods together for the addition and removal of all independent variables from the stepwise model depending upon the statistical performance [45]. The procedures of both models (like forward and backward) along with different variables are given in Figure 3 and Figure 4.

#### The Execution of SWLDA

SWLDA model starts with an empty model, meaning there are no variables for prediction at the beginning. Significance tests such as partial *F*-tests and *t*-tests are performed. Based on these tests, a predictor variable is either added or removed in each iteration. It also sets two threshold parameters, one is known as alpha-to-enter ($\alpha_{e}$) and the other is alpha-to-remove ($\alpha_{\gamma }$), for deciding the entry and removal of variables. Here, these threshold parameters are set as $\alpha_{e}$ = 0.35 and $\alpha_{\gamma }$ = 0.4. These threshold parameters also show the significance of the projected variable which is entered or removed through the model. The process continues until no more predictor variables might be passed in or passed out through the model.

Now let us assume, we have three independent variables, $C_{1}$, $C_{2}$, and $C_{3}$, and one output variable, *y*. We use regression to fit these variables inside the proposed model. Let ‘$P_{j}$’ indicate the number of projector variables. We use the recursion of *y* on $C_{1}$, the recursion of *y* on $C_{2}$, and so on until the recursion of *y* on $C_{p-1}$.

- **First Step**
  ○ The projector along with the least $P_{j}$-value, less than $\alpha_{e}$ = 0.35, from the *t*-test, will be the one to enter first in the proposed model.
  ○ This procedure will continue until the terminating criterion is reached, which is no variable having a $P_{j}$-value below $\alpha_{e}$.
  ○ Now suppose $C_{1}$ is the best projector that we find in the next step, we fix the endure projector method along with the best projector, $C_{1}$, in the model; i.e., recursion of *y* on ($C_{1}$, $C_{2}$), recursion of *y* on ($C_{1}$, $C_{3}$), and so on until the recursion of *y* on ($C_{1}$, $C_{p-1}$).

- **Second Step**
  ○ In the second step, the projector along with the least $P_{j}$-value ($\alpha_{e}$ = 0.35) is injected through the proposed model. Once more, the repetition terminates when no $P_{j}$-value is less than 0.35.
  ○ Let us suppose, that in this second iteration, $C_{2}$ is the “best second predictor” in the model. At this stage, the algorithm pulls back and examines the $P_{j}$-value for $\beta_{1}$ = 0, which indicates whether there is a need to remove the predictor variable from the model.
  ○ If the $P_{j}$-value of the projector variable is beyond $\alpha_{\gamma }$ = 0.4 for $\beta_{1}$ = 0, then that variable is not very important compared to the new one. However, if both the variables, $C_{1}$ and $C_{2}$, are selected through the two-projector proposed model, then the algorithm fits every one of the three-projector methods along with $C_{1}$ and $C_{2}$ in the method, like recursion of y on ($C_{1}$, $C_{2}$, $C_{3}$), recursion of y on ($C_{1}$, $C_{2}$, $C_{4}$), and so on until the recursion of y on ($C_{1}$, $C_{2}$, $C_{p-1}$).

- **Third Step**
  ○ The third predictor variable that injects the proposed model is the projector which has the least $P_{j}$-value (<$\alpha_{e}$ = 0.35).
  ○ This process again continues until the terminating criterion is reached, which is when there are no *p*-values less than $\alpha_{e}$. In this case, when we reach the stopping point, the algorithm examines the $P_{j}$-values for $\beta_{1}$ = 0.
  ○ If either of them is not significant anymore (above $\alpha_{\gamma }$ = 0.4), it removes that variable from the proposed model. The method terminates when adding more variables does not outcome in a $P_{j}$-value below $\alpha_{e}$ = 0.35.

### 3.4. Artificial Neural Network-Based (ANN) based Classification

**Figure 5:**
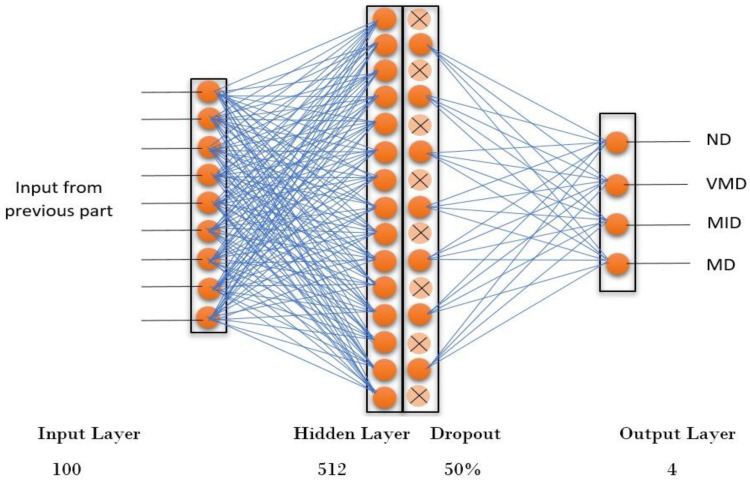
ANN architecture with fully connected layer and dropout for Alzheimer’s classification.

The ANN has three layers: the input layer, the hidden layer, and the output layer. The input layer has 150 nodes, corresponding to the 150 principal components (PCs) obtained from the principal component analysis (PCA) of the original features. The first 150 PCs account for more than 95% of the total variance, which means that they capture most of the relevant information from the MRI images while reducing the dimensionality and noise. The hidden layer has 512 nodes, with a dropout rate of 0.5 to prevent overfitting. The number of neurons in the hidden layer is chosen based on a heuristic that suggests using a value between the size of the input layer and the size of the output layer. The output layer has four nodes, representing the four classes of AD: non-demented (ND), mildly demented (MD), moderately demented (MOD), and very mildly demented (VMD). The activation function for the hidden layer is the rectified linear unit (ReLU), and the activation function for the output layer is the Softmax function, which produces a probability distribution over the four classes. The ANN is trained using the Adam optimizer and the categorical cross-entropy loss function (as shown in Figure 5).

The number of trainable parameters in the ANN is 79,364, and it is calculated as follows:

- Input layer to hidden layer, (100 × 512) + 512 = 51,712;
- Hidden layer to output layer, (512 × 4) + 4 = 2052;
- Dropout = 50%;
- Total, 51,712 + 2052 = 53,764.

The other parameters of the ANN are as follows:

- Batch size, 32;
- Number of epochs, 100;
- Learning rate, 0.001;
- Validation split, 0.25.

## 4. System Assessment

The suggested model was examined and verified on a standard brain MRI dataset of AD. The proposed system has been implemented in Python with the specification of 4 GB RAM and a 2.8 GHz processor. The system was assessed based on the following arrangements.

### 4.1. Brain MRI Dataset of AD

The dataset is a fascinating part of the image classification and pattern recognition domains. The major inspiration of this study is to suggest an ensembled framework for AD classification, the employed MRI dataset that was collected from numerous hospitals, public databases, and websites, which was generated by Sarvesh Dubey (Kaggle). There is a total of 6400 MRI images in this dataset that includes 3200 MRI slices for non-demented, 64 MRI slices for moderately demented, 896 MRI slices for mildly demented slices, and 2240 MRI slices for very mildly demented images. The format for the entire collection of MRI slices is JGP and the size for them during the experiments is 128 × 128 pixels.

### 4.2. Arrangements of Experiments

The system has been assessed and evaluated using successive sets of experimentation.

- In the first experiment, the accomplishment of the suggested hybrid approach against the brain MRI dataset is presented. For this purpose, we have selected a 10-fold out-of-sample testing rule (which was selected based on multiple experiments) for such an experiment. In this cross-validation scheme, the nine MRI images are exploited for training, while the last MRI image is used for testing. This process is repeated 10 times to ensure that every MRI image has been utilized for training and validation, respectively.
- The next experiment describes the significance of the MRI classification systems instead of using the suggested hybrid method. For this purpose, we exploited various combinations of machine learning methods to indicate the weightage of the suggested method.
- The last experiment indicates the comparison of the suggested approach against the latest existing systems using an open-brain MRI dataset. We compared the results in various conditions such as recognition rates, misclassification rates, true positive, true negative, false positive, false negative, sensitivity, and specificity against the brain MRI dataset.

## 5. Results and Discussions

### 5.1. First Experiment

This experiment presents the accuracy of the proposed hybrid approach against brain MRI. The entire performance is represented in Table 1.

**Figure 6:**
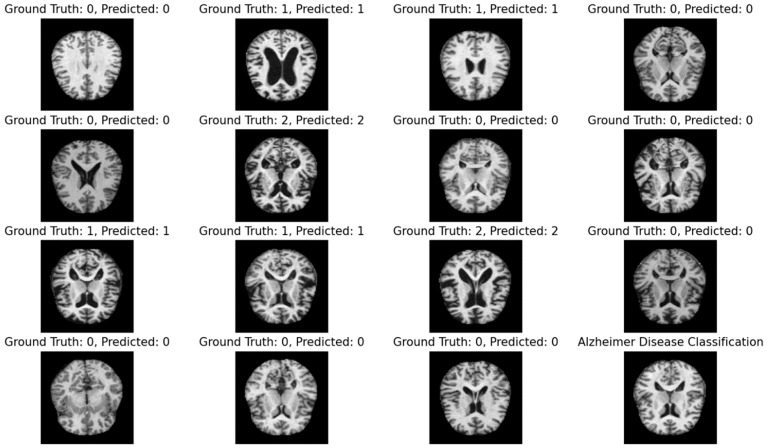
Predicted sample results of the proposed approach against the ground truth.

Table 1 illustrates that the suggested hybrid approach attained the best recognition rate using brain MRI. The suggested method selects only limited features through forward together with backward recursion methods. In the forward recursion method, the most interrelated features are identified based on the values of the partial *Z*-test while, in the backward recursion method, the slightest correlated features are removed from the same feature space. In both cases, the *Z*-test values are assessed using the provided disease labels. The proposed method has a notable strength in its ability to efficiently and effectively identify localized features. The predicted results against the ground truth are represented in Figure 6.

### 5.2. Second Experiment

This experiment evaluates the combination of various kinds of machine learning methods in the brain MRI classification system. The main goal of this evaluation is to show the importance of the proposed feature selection approach. Therefore, we employed different kinds of the latest methods of feature selection instead of utilizing the developed feature selection approach. We exploited random forest, k-nearest neighbor (kNN), decision tree, XGBoost, AdaBoost, light-gradient-boosting machine (LightGBM), CNN, PCA with ANN, and SVM. The results for each of the methods are represented in Table 2, Table 3, Table 4, Table 5, Table 6, Table 7, Table 8, Table 9 and Table 10.

As demonstrated in Table 2, Table 3, Table 4, Table 5, Table 6, Table 7, Table 8, Table 9 and Table 10, a proposed hybrid approach such as the developed feature selection method has a major role in achieving a high recognition rate in the brain MRI classification system of Alzheimer’s disease. When the suggested method is detached from the system, the classification rates are abruptly reduced. Table 2, Table 3, Table 4, Table 5, Table 6, Table 7, Table 8, Table 9 and Table 10 demonstrate the high level of similarity among the features of different diseases. The aforementioned results validate our investigation and offer valuable suggestions, and have allowed us to conclude that the suggested method chose the best set of features in terms of classification rate.

### 5.3. Third Experiment

Finally, this experiment presents a comparison of the suggested hybrid approach using the latest works. For some of the systems, we used the accuracies given in their reports; for other studies, we took their respective simulations. All of the studies have been executed in the exact environments prescribed in their respective reports. The comparisons are given in Table 11.

Table 11 demonstrates that the suggested hybrid methodology achieved the best classification rate against a state-of-the-art system on MRI images. This is because the suggested hybrid approach has the ability to choose the best features from the brain MRI. The corresponding selected features are employed for discriminating various disease classes by utilizing recursion methods like *Z*-values. In the suggested hybrid methodology, the forward model chooses only a set of features based on the specified criteria, while the backward model removes the most inappropriate features from the respective model. The values of the *Z*-test are evaluated in both models through the labels of the given diseases.

On the other hand, the proposed approach has been further tested and validated on other publicly available datasets such as the Alzheimer Parkinson Diseases 3 Class dataset [60] in order to demonstrate its robustness. The dataset contains three different types of diseases like Control, Alzheimer’s disease, and Parkinson’s disease, and contains a total of 7778 images. The overall results of the proposed approach against this dataset are presented in Table 12.

Table 12 demonstrates that the proposed hybrid approach not only demonstrated a performance compared to the single dataset but also achieved the better accuracy than the other dataset. These results indicate that the proposed hybrid approach is not only accurate but also robust across the datasets.

Furthermore, we have provided the comparison of the proposed approach against state-of-the-art systems using the Alzheimer Parkinson Diseases 3 Class dataset under the settings described in Section 4.2. The comparison results of the existing works along with the proposed approach with the Alzheimer Parkinson Diseases 3 Class dataset are presented in Table 13.

Table 13 demonstrates that the proposed hybrid approach also achieved the best recognition rate, compared to other existing works, with the Alzheimer Parkinson Diseases 3 Class dataset. This shows that the proposed approach is more robust than others.

### 5.4. Discussion

Exploring Alzheimer’s disease (AD) with adaptive machine learning algorithms has yielded promising outcomes. However, achieving a meaningful impact in medical settings requires a combination of high precision, reduced processing time, and applicability across diverse populations. This study introduces a hybrid and refined method for Alzheimer’s disease (AD) classification, utilizing a brain MRI dataset. Our approach incorporates a novel technique known as stepwise linear discriminant analysis (SWLDA), which efficiently identifies a limited set of features through a combination of forward and backward recursion methods. To demonstrate the significance of our developed approach, we utilized a publicly available brain MRI dataset comprising non-demented, mildly demented, moderately demented, and very mildly demented cases. We rigorously tested and validated our proposed method using this MRI dataset, dividing it into three sub-experiments.

The initial sub-experiment, depicted in Table 1, highlights that our hybrid approach achieved the highest recognition rate when applied to brain MRI data. This method employed both forward and backward recursion techniques. With the forward recursion method, it identifies the most interconnected features based on partial *Z*-test values. Conversely, with the backward recursion method, it eliminates the least correlated features from the same feature space. In both scenarios, the *Z*-test values are evaluated using the given disease labels. The proposed method demonstrates a significant capability in efficiently and accurately pinpointing specific features.

The second experiment, presented in Table 2, Table 3, Table 4, Table 5, Table 6, Table 7, Table 8, Table 9 and Table 10, underscores the significance of our proposed feature selection approach. Therefore, we opted to utilize various contemporary feature selection methods instead of our own developed approach. As depicted in Table 2, Table 3, Table 4, Table 5, Table 6, Table 7, Table 8, Table 9 and Table 10, the proposed feature selection method plays a pivotal role in achieving a high recognition rate in the Alzheimer’s disease classification system, using brain MRI data. When the suggested method is removed from the system, the classification rates undergo a sharp decline. Table 2, Table 3, Table 4, Table 5, Table 6, Table 7, Table 8, Table 9 and Table 10 reveal a consistent trend of high similarity among the features of different diseases. These findings corroborate our research and provide strong evidence that the suggested method effectively selects the best set of features in terms of classification rate.

Finally, we compared the proposed approach with state-of-the-art systems using a brain MRI dataset, as presented in Table 11. All of the systems were implemented following the exact settings described in their respective articles. It is evident that the proposed approach exhibited remarkable performance compared to existing works.

## 6. Conclusions

Alzheimer’s disease (AD) stands out as one of the most formidable conditions, necessitating early and precise diagnosis to facilitate effective treatment strategies. In this study, we have proposed a hybrid approach model for the categorization of AD using an MRI dataset. In this work, at the initial stage, during the preprocessing phase, we integrated an averaging filter to diminish irrelevant or excessive details. Following that, we employed a combined approach, which involved the use of principal component analysis (PCA) along with stepwise linear discriminant analysis (SWLDA), followed by the utilization of an artificial neural network (ANN). The goal of this approach was to extract and identify the most effective features, streamlining the process of classifying various diseases using MRI data. SWLDA utilizes a combination of forward and backward recursion techniques to select a limited set of features. The forward recursion identifies the most interconnected features by analyzing partial Z-test values, while the backward recursion method eliminates the least correlated features from the same feature space. Following feature extraction and selection, we employed an optimized artificial neural network (ANN) to distinguish different classes of Alzheimer’s disease. The proposed approach was tested and validated on a publicly available standard brain MRI dataset, which showed a weighted average recognition rate of 99.35% against state-of-the-art systems.

Within healthcare fields, certain systems may become necessary tools for physicians to achieve precise Alzheimer’s disease classification. Consequently, we intend to implement the suggested approach in healthcare domains in the near future, to assist healthcare professionals. The proposed approach is tested and validated on a small set of sample data; therefore, in future, we will also assess the comprehensive performance of the proposed system by analyzing a substantial sample size. Furthermore, we will also investigate the feasibility of employing various deep learning neural networks in a broader context.

## Figures and Tables

**Table 1:** Rates of classification of the suggested hybrid approach using MRI images: ND—non-demented; MD—moderately demented; MID—mildly demented; VMD—very mildly demented.

	Recognition Rates (%)				
**Proposed Hybrid Approach**	**Diseases**	**ND**	**VMD**	**MID**	**MD**
	**ND**	99.4	0.5	0.1	0
	**VMD**	1.5	98.0	0.3	0
	**MID**	0.9	1.2	100	0
	**MD**	0	0	0	100
	**Average**	**99.35**			

**Table 2:** Classification rates of the random forest against MRI dataset: ND—non-demented; MD—moderately demented; MID—mildly demented; VMD—very mildly demented.

	Recognition Rates (%)				
**Proposed Hybrid Approach**	**Diseases**	**ND**	**VMD**	**MID**	**MD**
	**ND**	99.0	1	0	0
	**VMD**	11.8	88.2	0	0
	**MID**	8.4	10.9	80.7	0
	**MD**	16.6	16.6	0	66.8
	**Average**	**83.68**			

**Table 3:** Classification rates of the k-nearest neighbor (kNN) against MRI dataset: ND—non-demented; MD—moderately demented; MID—mildly demented; VMD—very mildly demented.

	Recognition Rates (%)				
**Proposed Hybrid Approach**	**Diseases**	**ND**	**VMD**	**MID**	**MD**
	**ND**	98.9	0.6	0.5	0
	**VMD**	3.6	96.4	0	0
	**MID**	2	1.5	96.5	0
	**MD**	0	4.2	4.2	91.6
	**Average**	**95.85**			

**Table 4:** Classification rates of decision tree against MRI dataset: ND—non-demented; MD—moderately demented; MID—mildly demented; VMD—very mildly demented.

	Recognition Rates (%)				
**Proposed Hybrid Approach**	**Diseases**	**ND**	**VMD**	**MID**	**MD**
	**ND**	75.2	16.6	8.0	0.2
	**VMD**	21.6	67.1	10.9	0.4
	**MID**	14.2	24.0	61.8	0
	**MD**	8.3	29.2	12.5	50.0
	**Average**	**71.02**			

**Table 5:** Classification rates of XGBoost against MRI dataset: ND—non-demented; MD—moderately demented; MID—mildly demented; VMD—very mildly demented.

	Recognition Rates (%)				
**Proposed Hybrid Approach**	**Diseases**	**ND**	**VMD**	**MID**	**MD**
	**ND**	99.5	0.5	0	0
	**VMD**	3.4	96.6	0	0
	**MID**	0.5	4.0	95.5	0
	**MD**	12.5	37.5	0	50.0
	**Average**	**85.4**			

**Table 6:** Classification rates of the AdaBoost against MRI dataset: ND—non-demented; MD—moderately demented; MID—mildly demented; VMD—very mildly demented.

	Recognition Rates (%)				
**Proposed Hybrid Approach**	**Diseases**	**ND**	**VMD**	**MID**	**MD**
	**ND**	75.8	19.7	4.5	0
	**VMD**	49.7	42.5	7.8	0
	**MID**	38.8	42.3	18.9	0
	**MD**	20.9	33.3	12.5	33.3
	**Average**	**42.63**			

**Table 7:** Classification rates of the light-gradient-boosting machine (LightGBM) against MRI dataset: ND—non-demented; MD—moderately demented; MID—mildly demented; VMD—very mildly demented.

	Recognition Rates (%)				
**Proposed Hybrid Approach**	**Diseases**	**ND**	**VMD**	**MID**	**MD**
	**ND**	99.6	0.4	0	0
	**VMD**	2.9	97.1	0	0
	**MID**	0	5.0	95.0	0
	**MD**	4.2	29.2	0	66.6
	**Average**	**89.58**			

**Table 8:** Classification rates of CNN against MRI dataset: ND—non-demented; MD—moderately demented; MID—mildly demented; VMD—very mildly demented.

	Recognition Rates (%)				
**Proposed Hybrid Approach**	**Diseases**	**ND**	**VMD**	**MID**	**MD**
	**ND**	98.2	1.2	0.6	0
	**VMD**	3.5	95.3	1.2	0
	**MID**	3.1	7.0	89.9	0
	**MD**	1.7	9.5	11.3	77.4
	**Average**	**90.20**			

**Table 9:** Classification rates of ANN against MRI dataset: ND—non-demented; MD—moderately demented; MID—mildly demented; VMD—very mildly demented.

	Recognition Rates (%)				
**Proposed Hybrid Approach**	**Diseases**	**ND**	**VMD**	**MID**	**MD**
	**ND**	93.7	3.7	2.6	0
	**VMD**	6.8	88.4	4.2	0.6
	**MID**	1.5	2.4	96.1	0
	**MD**	3.1	12.3	1.1	83.5
	**Average**	**90.42**			

**Table 10:** Classification rates of the SVM against MRI dataset: ND—non-demented; MD—moderately demented; MID—mildly demented; VMD—very mildly demented.

	Recognition Rates (%)				
**Proposed Hybrid Approach**	**Diseases**	**ND**	**VMD**	**MID**	**MD**
	**ND**	95.4	2.3	1.3	1.0
	**VMD**	7.8	85.6	6.6	0
	**MID**	11.2	8.4	71.6	8.8
	**MD**	2.3	18.5	9.3	69.9
	**Average**	**80.62**			

**Table 11:** Comparison of the proposed hybrid approach for Alzheimer’s disease identification using MRI dataset.

Existing Approaches	Utilized Methods	Accuracy	Misidentification
Isunuri et al. [47]	Transfer learning with residual convolution network	97.3%	2.7%
Francisco Santos [48]	Convolutional neural networks (CNN)	80.6%	19.4%
Falco et al. [49]	Interpretable machine learning (IML)	91.4%	8.6%
Priyatama et al. [50]	CNN, VGG-16, and VGG-19	80.2%	19.8%
Kim, R [51]	Hybrid quantum-classical neural network	93.0%	7.0%
Liu et al. [52]	Multi-phantom convolution (MPC), space conversion attention mechanism (MPC-STANet), and ResNet50	94.1%	5.9%
Sharma et al. [53]	Transfer learning (TL)	94.9%	5.1%
Karakaya et al. [54]	Deep CNN	95.7%	4.3%
Sharma et al. [55]	TL and permutation-based ML	91.7%	8.3%
Hao et al. [56]	CNN	86.8%	13.2%
El-Latif et al. [57]	Lightweight deep learning model	95.9%	4.1%
Subramoniam et al. [58]	TL, VGG, and ResNet	85.1%	14.9%
Murugan et al. [59]	CNN and DEMentia NETwork	93.0%	7.0%
**Proposed Hybrid Approach**	**PCA, SWLDA, and ANN**	**99.35%**	**0.65%**

**Table 12:** Rates of classification of the suggested hybrid approach using Alzheimer Parkinson Diseases 3 Class dataset.

	Recognition Rates (%)			
**Proposed Hybrid Approach**	**Disease**	**Control**	**Alzheimer’s Disease**	**Parkinson’s Disease**
	**CONTROL**	97.2	0.4	2.4
	**Alzheimer’s disease**	0.3	99.7	0
	**Parkinson’s disease**	0	6.9	93.1
	**Average**	**96.66**		

**Table 13:** Comparison of the proposed hybrid approach with Alzheimer Parkinson Diseases 3 Class dataset.

Existing Approaches	Utilized Methods	Accuracy	Misidentification
Isunuri et al. [47]	Transfer learning with residual convolution network	93.4%	6.6%
Francisco Santos [48]	Convolutional neural networks (CNN)	77.8%	22.2%
Falco et al. [49]	Interpretable machine learning (IML)	87.9%	12.1%
Priyatama et al. [50]	CNN, VGG-16, and VGG-19	82.5%	17.5%
Kim, R [51]	Hybrid quantum-classical neural network	89.9%	10.1%
Liu et al. [52]	Multi-phantom convolution (MPC), space conversion attention mechanism (MPC-STANet), and ResNet50	90.5%	9.5%
Sharma et al. [53]	Transfer learning (TL)	92.4%	7.6%
Karakaya et al. [54]	Deep-CNN	94.3%	5.7%
Sharma et al. [55]	TL and permutation-based ML	86.7%	13.3%
Hao et al. [56]	CNN	88.1%	11.9%
El-Latif et al. [57]	Lightweight deep learning model	90.3%	9.7%
Subramoniam et al. [58]	TL, VGG, and ResNet	80.9%	19.1%
Murugan et al. [59]	CNN and DEMentia NETwork	89.5%	10.5%
**Proposed Hybrid Approach**	**PCA, SWLDA, and ANN**	**96.6%**	**3.4%**

## Data Availability

The Alzheimer’s Dataset utilized in this research is publicly available: https://www.kaggle.com/tourist55/alzheimers-dataset-4-class-of-images (accessed on 5 February 2023) and https://www.kaggle.com/datasets/farjanakabirsamanta/alzheimer-diseases-3-class (accessed on 6 September 2023).
